# Population-Level Trends in Lifestyle Factors and Early-Onset Breast, Colorectal, and Uterine Cancers

**DOI:** 10.3390/cancers18010167

**Published:** 2026-01-03

**Authors:** Natalie L. Ayoub, Alex A. Francoeur, Jenny Chang, Nathan Tran, Krishnansu S. Tewari, Daniel S. Kapp, Robert E. Bristow, John K. Chan

**Affiliations:** 1 Department of Gynecologic Oncology, University of California Irvine, Orange, CA 92868, USA; 2 Department of Gynecologic Oncology, Sutter Health, Walnut Creek, CA 94598, USAjohn.chan@sutterhealth.org (J.K.C.); 3 Department of Radiation Oncology, Stanford University, Stanford, CA 94304, USA; dskapp@stanford.edu

**Keywords:** early-onset cancers, cancer incidence, breast cancer, colorectal cancer, uterine cancer, obesity, modifiable risk factors

## Abstract

**Objective:** To evaluate population-level temporal relationships between modifiable lifestyle factors and rising breast, colorectal and uterine cancer incidence rates among females under 50 years old. **Methods:** This retrospective ecological study utilized data from the United States Cancer Statistics (USCS) for cancer incidence, the National Health and Nutrition Examination Survey (NHANES) for health-related behaviors, and the Behavioral Risk Factor Surveillance System (BRFSS) for physical activity. Modifiable lifestyle factors analyzed included obesity (BMI ≥ 30 kg/m^2^), smoking, alcohol use, fiber and saturated fat intake, caloric intake, and physical activity. Trends were assessed using average annual percent change (AAPC), and population-level correlations between cancer incidence and lifestyle factors were evaluated using Pearson correlation coefficients. **Results:** Between 2001 and 2018, 914,659 breast, 144,130 colorectal, and 124,399 uterine cancer cases were identified. The largest increases in cancer incidence occurred in age groups under 30 years old. Colorectal cancer increased by 6.9%, followed by uterine cancer at 4.8% and breast cancer at 1.7%, all *p* < 0.001. When examining this age group by race, colorectal cancer increased by 8.0% (*p* < 0.001) annually in White women aged 20–24 years, while uterine cancer rose 4.8% (*p* < 0.001) in Hispanic women in the 20–24 and 25–29 year age groups. Breast cancer also increased by 2.0% (*p* < 0.001) per year in White women 25–29 years old. Smoking rates decreased, and alcohol consumption and obesity rates increased. No significant correlation was found between cancer incidence and smoking, caloric intake, saturated fat, or physical activity. A moderate positive correlation was identified between alcohol use and cancer risk (r = 0.55–0.67, *p* < 0.05). Obesity prevalence showed strong population-level temporal correlation with cancer incidence for all three cancers with stratified analysis demonstrating the strongest correlations in patients with class III obesity. **Conclusions:** From 2001 to 2018, the incidence of breast, colorectal, and uterine cancers increased most sharply among women under 30 years of age. Over the same period, obesity prevalence in this population also increased. These population-level observations are hypothesis-generating and require confirmation in individual-level, prospective studies to determine whether and how obesity and other lifestyle factors influence early-onset cancer risk.

## Simple Summary

Cancer diagnoses in women younger than 50 years old are rising in the United States, including breast, colorectal, and uterine cancers. At the same time, obesity has become more common in young adults. Understanding whether these trends are occurring together may help guide prevention and early detection strategies. In this study, we used national cancer registry data (2001–2018) and national health survey data from the same years to describe how cancer rates and lifestyle factors changed over time in women aged 20–49 years. We found that these cancers increased most sharply in women under 30 years of age, and obesity prevalence also rose over the same period. These findings highlight an important public health concern and support the need for future individual-level studies to clarify drives of early-onset cancer and inform targeted prevention efforts.

## 1. Introduction

Over the past twenty years, the incidence of breast, uterine and colorectal cancers have been increasing at a rate of 1–2% annually in women aged 20–49 years [1]. Specifically, both early- and late-stage colorectal cancer diagnoses have significantly increased among non-Hispanic White (White) and Hispanic populations, while breast cancer rates have similarly risen by 1.4% annually in women under 50 [2,3]. Multiple population-based studies have also demonstrated an increased incidence in uterine cancer in young, reproductive age women [4,5,6].

Colorectal screening was only recently lowered to begin at age 45, despite data showing rising incidence in younger adults [7]. For breast cancer, screening recommendations vary, but younger women often face barriers to early detection, particularly in racial minority groups [8]. Addressing these disparities and identifying modifiable risk factors is crucial to reversing the rising burden of cancer in younger populations. Excess body weight is a modifiable risk factor strongly linked to breast, colorectal, and uterine cancers [9,10]. Given their established links to adiposity and their increasing burden in younger populations, they serve as important models for exploring the relationship between obesity and early-onset cancer risk [11,12].

The objectives of this study were to evaluate the proportion of breast, colorectal and uterine cancers in young females, to examine trends in the incidence of these cancers over the past two decades, and to analyze the impact of lifestyle factors associated with cancer risk in this population.

## 2. Methods

### 2.1. Data Sources

This study used an ecological design, analyzing population-level data, which were obtained from three sources: the United States Cancer Statistics (USCS), National Health and Nutrition Examination Survey (NHANES), and the Behavioral Risk Factor Surveillance System (BRFSS). Because this was an ecological study, all analyses were based on aggregated population-level data, and individual-level associations could not be assessed. Deidentified cancer incidence data were obtained from the USCS database for patients diagnosed with cancer aged 20–49 years inclusive of the years 2001 to 2018. The study period was selected to account for the latency period between obesity and cancer development [13]. The USCS data source provides population-based data for the entirety of the United States and Puerto Rico, including over 37 million cancer cases [14]. The database is updated yearly, reflecting the most up-to-date and accurate information on cancer diagnoses in the United States [14]. The dataset includes age-adjusted incidence rate for age 20–49 and age-specific incidence (ASI) rates, expressed per 100,000 female individuals, for uterine, breast and colorectal cancers. For some age groups, case numbers were too low to meet reporting thresholds, resulting in unavailable data.

To evaluate ecologic correlation between sociodemographic and health characteristics and the cancer incidence in young females, modifiable lifestyle data were extracted from the NHANES database from 2001 to 2018 [15]. The following variables were extracted from the NHANES data source: obesity, smoking status, alcohol consumption, fiber intake, and total caloric intake [15]. Obesity was measured using body mass index (BMI, kg/m^2^) and subsequently stratified into three classes: Class I (BMI 30.0–34.9), Class II (BMI 35.0–39.9), and Class III (BMI ≥ 40.0). Smoking and alcohol use were coded as the average number of cigarettes smoked per day in the last 30 days, and average number of drinks per day in the past 12 months, respectively. Saturated fat, fiber (in grams), and total kilocalories were collected based on self-reported 24 hour dietary recall. Data from multiple NHANES survey cycles from the years 2001 through 2018 were aggregated to align with the cancer incidence years in USCS, allowing for trend comparisons. NHANES measurements, collected biennially, were applied equally to both years of each cycle when calculating rates. All NHANES data were analyzed using the provided survey weights to account for the complex, multistage probability sampling design and to produce national representative estimates. Unweighted sample sizes for female BMI measurements within five year age strata ranged from approximately 180 to 300 participants per survey cycle, supporting the stability of age-specific prevalence estimates (Supplementary Table S3).

BRFSS from 2001 to 2018 was used to abstract data on physical activity in females aged 20–49 [16]. This registry conducts cross-sectional telephone surveys on a monthly basis to collect data on preventive health practices of United States residents [16]. This system interviews more than 500,000 adult interviews each year, making it a qualified and comprehensive health survey in the United States. The survey inquired if participants participated in any physical activities or exercises in the past month. The results were reported as the percentage of respondents that participated in physical activity within the past month [16].

The analyses were stratified by self-reported race and age group to examine trends in early-onset cancers. Race/ethnicity were categorized as Non-Hispanic White (White), Non-Hispanic Black (Black), and Hispanic. Age groups were divided into 5 year increments: 20–24, 25–29, 30–34, 35–39, 40–44, and 45–49 years old based on USCS [14].

### 2.2. Statistical Analysis

Annual age-adjusted cancer incidence rates for age 20–49, along with age-specific 5-year incremental rates for cancer sites, were presented. Average annual percent change (AAPC) was calculated to assess temporal trends in cancer incidence during the study period, with 95% confidence intervals (CIs) and *p*-values reported to determine statistical significance. Temporal trends were analyzed using the Joinpoint Regression Program (version 5.4.0; Statistical Methodology and Applications Branch, Surveillance Research Program, National Cancer Institute). The number of joinpoints (segments) was selected using the Monte Carlo permutation test implemented in the software. Annual percent change (APC) was estimated for each segment, and the AAPC was calculated as a weighted average of segment-specific APCs, with weights equal to the length of each segment over the specified interval.

To evaluate potential collinearity trends between modifiable lifestyle factors and cancer incidence trends, Pearson correlation coefficients were computed between cancer rate for breast, colorectal and uterine cancer incidence and NHANES or BRFSS risk factors for the same age and racial group. This analysis aimed to identify potential lifestyle contributors to the observed rise in early-onset cancers. Correlations were assessed for statistical significance, with *p*-values < 0.05 considered significant. All statistical analyses were conducted using SAS 9.4 (SAS Institute Inc., Cary, NC, USA) and Joinpoint Regression Program (version 5.4.0). Data visualizations, including trend graphs and scatter plots, were generated to illustrate relationships between cancer incidence rates and modifiable risk factors. All of the data were derived from de-identified records, and thus no institutional review board approval was required. This study followed Strengthening the Reporting of Observational studies in Epidemiology (STROBE) reporting guidelines [17].

## 3. Results

### 3.1. Study Population

A total of 4,868,268 breast, 1,423,948 colorectal, and 996,362 uterine cancer cases were identified across all age groups, 20 to 85 years and older, between 2001 and 2018. Among women aged 20–49 years old, there were 914,659 breast, 144,130 colorectal, and 124,399 uterine cancer cases identified (Supplementary Table S1). In the breast cancer cohort, women aged 20–24 accounted for 0.07% of cases, followed by 0.44% in patients 25–29 years, 1.31% in 30–34, 2.86% in 35–39 years, 5.81% in 40–44, and 9.03% in the 45–49 age group. In the colorectal sample, women aged 20–24 comprise 0.21% of cases, followed by those aged 25–29 (0.41%), 30–34 (0.80%), 35–39 (1.46%), 40–44 (2.64%), and 45–49 (4.60%). Lastly, in the uterine cancer group, patients 20–24 years old made up 0.10% of cases, 25–29 (0.41%), 30–34 (1.05%), 35–39 (1.98%), 40–44 (3.34%), and 45–49 (5.61%) (Supplementary Table S1).

### 3.2. Average Annual Percent Change by Disease Site

Breast cancer rates from 2001 to 2018 revealed a significant increase in AAPC for women 20–49 years old (AAPC 0.44%, 95% CI 0.30–0.60; *p* < 0.001), and for each prespecified age group. The greatest increase occurred in patients 20–24 and 25–29 years old, with a 1.69% (95% CI 1.0–2.4; *p* < 0.001) and 1.62% (95% CI 1.20–2.10; *p* < 0.001) annual growth rate, respectively. Colorectal cancer rates also increased in the overall study population (AAPC 1.75%; 95% CI 1.4–2.1; *p* < 0.001); however, the greatest rate of increase was in women aged 20–24 at 6.92% (95% CI: 4.3–9.6; *p* < 0.001), and 25–29 years old at 4.15% (95% CI 3.2–5.2; *p* < 0.001). Lastly, uterine cancer has been increasing significantly in all patients between 20 and 49 years old at a rate of 1.64% annually, (95% CI 1.4–1.9; *p* < 0.001), however, young patients aged 25–29 (AAPC 4.80%; 95% CI 1.8–7.8, *p* < 0.01) and 30–34 years old (AAPC 4.78%; 95% CI 3.9–5.7; *p* < 0.001) had the greatest increases in average yearly growth rate (Table 1).

### 3.3. Race/Ethnicity Analysis

**Figure 1:**
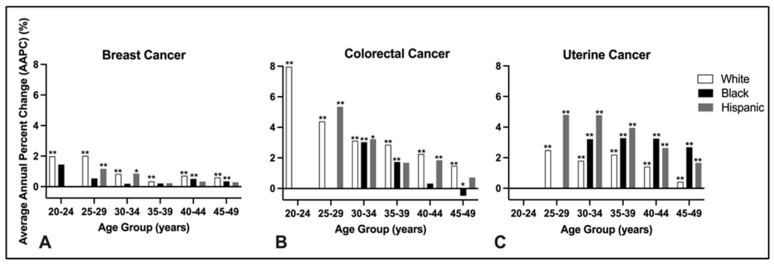
Average Annual Percent Change (AAPC) in cancer incidence by age group and race among females aged 20–49 years. Bar graphs display the AAPC in incidence of (**A**) breast, (**B**) colorectal, and (**C**) uterine cancers from 2001 to 2018, stratified by age group and race (White, Black, and Hispanic). Data extracted from USCS database. Asterisks indicated AAPC values significantly different from zero within each subgroup (* *p* < 0.05, ** *p* < 0.01; Joinpoint regression).

Overall mean annual percent change was calculated for each racial group and cancer type. Due to extremely low case counts in certain age groups, particularly among women aged 20–24, race-specific data could not be calculated for all racial groups (Table 1). Accordingly, race and ethnicity-stratified findings should be interpreted as descriptive trend estimates and may be unstable in strata with small case counts. The annual percent change in breast cancer incidence among women aged 20–49 years old was 0.62% (95% CI 0.5–0.8; *p* < 0.001) in White and 0.31% (95% CI 0.1–0.5; *p* < 0.001) in Black women (Table 1). The average yearly percent change in colorectal cancer incidence among women of the same age group was 2.27% (95% CI 1.8–2.8; *p* < 0.001) in White women (Table 1). The AAPC for uterine cancer among the entire study population aged 20–49 years old could not be calculated by race within the uterine cancer subgroup due to incomplete data in certain age categories (Table 1). When examining the data by five-year age groups and race, White females between 25 and 29 years old had the largest increase in breast cancer incidence, rising on average by 2.02% per year (95% CI 1.3–2.8; *p* < 0.001) (Figure 1A). For colorectal cancer, the highest increases were seen among White individuals between 20 and 24 years, with an average annual growth rate of 7.97% (95% CI 4.6–11.4; *p* < 0.001) and Hispanic individuals aged 25–29, with an increase of 5.35% per year (95% CI 2.7–8.1; *p* < 0.001) (Figure 1B). Lastly, in uterine cancer, Hispanic females aged 25–29 and 30–34 had the most pronounced increases, with annual growth rates of 4.80% (95% CI 1.8–7.8; *p* < 0.001) and 4.78% (95% CI 3.9–5.7; *p* < 0.001), respectively (Figure 1C).

### 3.4. Modifiable Lifestyle Risk Factors

Using the NHANES dataset we examined population trends in obesity, smoking status, alcohol consumption, fiber intake, saturated fat intake, and total caloric intake during the study period. Obesity rates for the entire study population aged 20–49 years old increased by an average of 1.49% every year (95% CI 0.9–2.1; *p* < 0.001) (Supplementary Figure S1A). The data were then divided by obesity class showing an average yearly percent change of 0.10% for patients with class I obesity (95% CI −0.8–1.1; *p* = 0.80), 1.63% for class II obesity (95% CI −0.8–4.0; *p* = 0.16), and 4.18% for class III obesity (95% CI 2.10–6.30; *p* < 0.001) (Supplementary Figure S1B). There was a decrease in average annual smoking rates by 4.84% (95% CI −6.7, −2.9; *p* < 0.001) and an increase in the number of alcoholic drinks (AAPC 1.45%; 95% CI 0.4–2.5; *p* = 0.005) (Table 2). There was an increase in the grams of fiber consumed (AAPC 0.97%; 95% CI 0.0–1.9; *p* = 0.05) (Supplementary Table S2). There was no change in kilocalories, saturated fat in grams or physical activity across the prespecified time period in women aged 20–49 years old (Supplementary Table S2).

### 3.5. Obesity and Cancer Incidence Correlation

**Figure 2:**
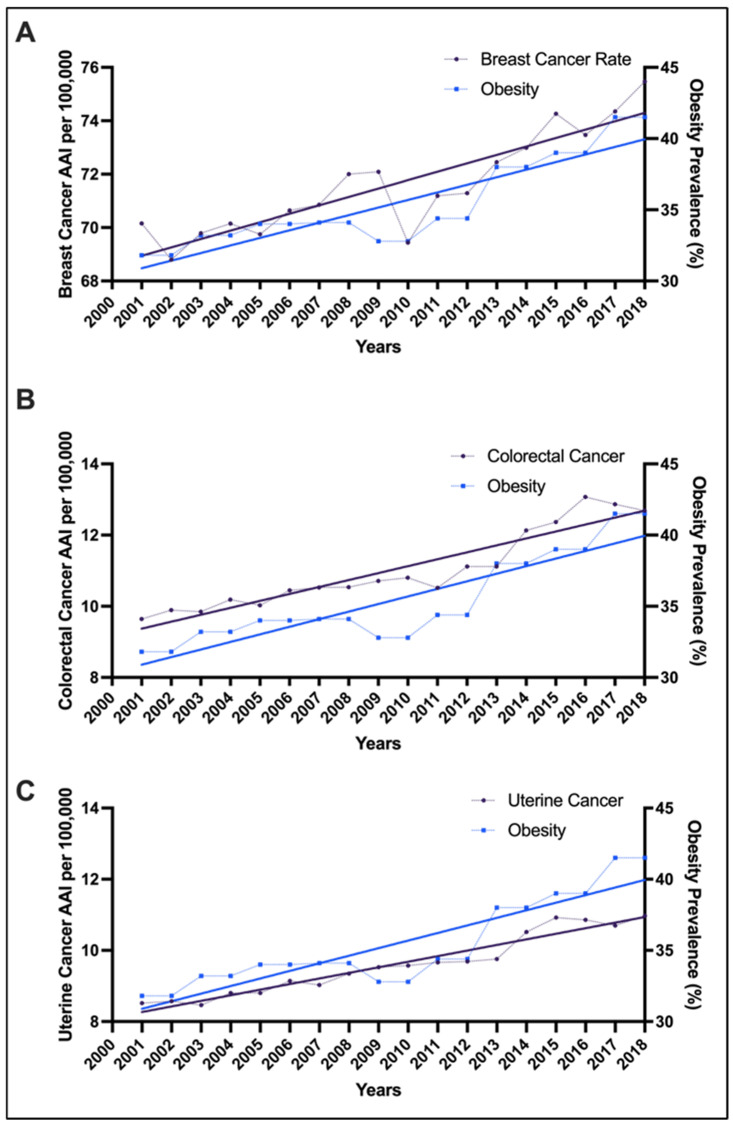
Trends in cancer incidence and obesity prevalence among females aged 20–49 years from NHANES (2001–2018). Line graphs depict Age-Specific Incidence (ASI) rates per 100,000 and Pearson correlation coefficients (r) for (**A**) breast (r = 0.92; *p* < 0.001), (**B**) colorectal (r = 0.93; *p* < 0.001), and (**C**) uterine cancer (r = 0.90; *p* < 0.001) (left y-axis) alongside obesity prevalence (BMI ≥ 30) as a percentage (right y-axis). Solid lines represent fitted linear trends across calendar years, while points indicate observed annual values. Across all three cancer types, incidence rates rose in parallel with increasing obesity rates.

**Figure 3:**
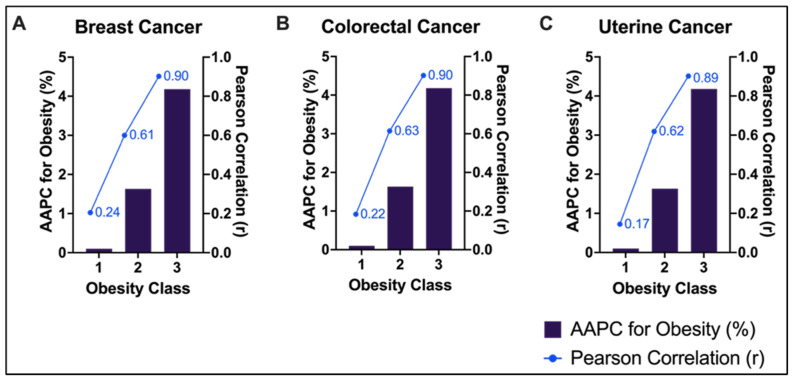
Graded association in aggregate trends between obesity class and cancer incidence in women aged 20–49 years from USCS and NHANES. Bar graphs show the AAPC in obesity prevalence for each obesity class for (**A**) breast, (**B**) colorectal and (**C**) uterine cancers. Overlaid line plots depict Pearson correlation coefficients between obesity and cancer incidence. A graded association between obesity severity and rising cancer incidence is depicted.

Pearson correlation analyses were performed to examine the relationship between trends in cancer, and the NHANES behavioral data. The yearly increase in breast (r = 0.55; *p* = 0.02), colorectal (r = 0.61; *p* = 0.007), and uterine cancer (r = 0.67; *p* = 0.0019) cases, was moderately correlated with the increase in alcohol consumption. There was a positive correlation in temporal trends between obesity (BMI ≥ 30) and increasing incidence of all three cancer types in patients aged 20–49 years for breast (r = 0.92; *p* < 0.001), colorectal (r = 0.93; *p* < 0.001) and uterine cancer (r = 0.90; *p* < 0.001) (Figure 2A–C). We then sought to examine the collinearity between obesity (BMI ≥ 30) and cancer incidence, stratified age group for each cancer type. Among all age groups, the 25–29 cohort showed that obesity and cancer incidence rose in parallel for breast cancer (r = 0.80; *p* < 0.001) and colorectal (r = 0.84; *p* < 0.001) cancers. In patients with uterine cancer, the strongest collinearity between obesity prevalence and the annualized rate of change was observed in the 35–39 age group (r = 0.78; *p* < 0.01), approximately a decade older than the peak collinearity seen in the other two cancer types. A stratified trend analysis demonstrated a statistically significant, graded collinearity between increasing obesity class and incidence of each of these three cancer types, with the strongest correlations seen in patients with class III obesity: breast (r = 0.90; *p* < 0.001), colorectal (r = 0.90; *p* < 0.001) and uterine cancer (r = 0.89; *p* < 0.001) (Figure 3A–C). Comparatively, the class I obesity cohort showed a weak correlation between obesity and mean annual percent change: breast (r = 0.24; *p* = 0.35), colorectal (r = 0.22; *p* = 0.38), uterine (r = 0.17; *p* = 0.50) (Figure 3A–C). We also further investigated the potential latency effect of obesity by assessing obesity prevalence during 2001–2009 in relation to cancer incidence during 2010–2018, the results revealed positive correlation for colorectal cancer (r = 0.79, *p* = 0.0119) and uterine cancer (r = 0.71, *p* = 0.0315) and a borderline correlation for breast cancer (r = 0.66, *p* = 0.0534).

## 4. Discussion

### 4.1. Summary

The rising incidence of breast, colorectal, and uterine cancers in young women represents a concerning shift in cancer epidemiology that warrants urgent attention. The data demonstrate the largest increases in cancer incidence are observed in women under 30 years old. There is a strong, monotonic population-level temporal trend between obesity and cancer risk across all three cancer types. In contrast, no significant associations were observed between cancer incidence and other lifestyle factors such as caloric intake, alcohol consumption, saturated fat, or physical activity, highlighting obesity as a prominent population-level trend occurring concurrently with rising early-onset cancer incidence.

### 4.2. Rising Early-Onset Cancer Rates

Multiple studies have shown increasing rates of colorectal, breast, and uterine cancer rates in adults under 50 years old [1,5,18,19,20,21]. Lifestyle and metabolic factors, particularly obesity, have been strongly implicated [22]. In women with cancer, obesity has been associated with a 62% increased risk of cancer-related death compared to those of normal weight (RR 1.62; 95% CI 1.4–1.87) [22]. Data from the POSH study in the United Kingdom (UK) showed that obese patients with breast cancer were more likely to present with larger tumors and lower disease-free and overall survival [23]. Data on colorectal and uterine cancer data are similar. The Nurses’ Health Study II demonstrated a stepwise increase in colorectal cancer risk per 5-unit increment in BMI, with weight gain greater than 40 kg conferring a 115% higher risk [24]. Obesity is a well-established risk factor for endometrioid endometrial cancer [5,25,26,27]. A large retrospective study found rising obesity rates in females less than 45, and a decline in mean endometrial cancer diagnosis age from 64 to 61 years [28]. These studies support the notion that rising obesity rates contribute to the increasing incidence of early-onset breast, colorectal and uterine cancers in young females and that risk is correlated to the degree of obesity. However, it is important to acknowledge that observed incidence increases may also reflect changes in screening practices and clinical awareness over time. These effects may differ by cancer type and age group and should be considered when interpreting secular trends. A definitive correlation from our study cannot be established due to the use of independent datasets. However, the observed trends are consistent with and support our hypothesis.

### 4.3. Lifestyle Factors Beyond Obesity

There is a growing body of evidence that suggests obesity may be a key driver of rising cancer rates; however, the biological mechanisms linking obesity to cancer risk are not well understood [25,29,30]. There are many hypotheses such as increased body adiposity being associated with higher levels of proinflammatory cytokines, hyperinsulinemia, excess production of steroid hormones, and changes to the gut microbiome [11,29,30]. This can increase circulating estrogen and leptin levels, which have been shown to promote tumor development by enhancing cell proliferation, supporting angiogenesis, and inhibiting apoptosis, particularly in hormonally driven cancers such as breast and endometrial cancer [31,32,33]. Given these findings, interventions that target weight reduction may reduce the risk of obesity-associated cancers. Bariatric surgery has been associated with significant reductions in cancer incidence, particularly for endometrial and postmenopausal breast cancers [26]. Similarly, Glucagon-Like Peptide (GLP-1) receptor agonists have shown promise in lowering cancer risk by promoting sustained weight loss and potentially modulating cancer-related metabolic pathways [27]. In addition to these clinical interventions, evidence-based lifestyle medicine approaches, including adequate sleep and stress reduction, also contribute to improved metabolic health and reductions in obesity related cancer risk [34,35,36]. Environmental and policy-level strategies that address obesogenic environments may further support population-wide prevention efforts [37,38]. Physical inactivity, smoking and alcohol consumption are also well-established risk factors for cancer [39,40,41]. In our study, we observed a decrease in smoking rates and an increase in alcohol consumption over time, but no significant changes in physical activity. No consistent population-level temporal correlation was observed between these lifestyle factors and cancer incidence trends in this ecological analysis. Importantly, the absence of population-level correlation between lifestyle risk factors and cancer incidence should not be interpreted as evidence against established individual-level associations reported in cohort studies and meta-analyses.

Dietary habits, such as low fiber intake and excess calories have also been associated with increased cancer risk, particularly for colorectal and breast cancers [42,43]. Despite these known associations, this study did not detect any significant trends in fiber intake, total calorie consumption or saturated fat intake. This discrepancy may be due to limitations in the NHANES dataset, which relies on self-reported food frequency data that may not capture long-term dietary patterns accurately.

### 4.4. Future Directions

These findings should be interpreted as hypothesis-generating and require confirmation in individual-level, prospective studies. Future directions should leverage a more comprehensive database with detailed information on diagnosis, treatment, and lifestyle variables to refine risk assessments and better account for confounding factors. Future analyses should also incorporate potential modifying risk factors, such as, diabetes, metabolic syndrome, hormonal contraception, and parity, to better understand their influence on cancer risk. Linking cancer incidence trends to county-level indicators such as socioeconomic measures or food environment data may further clarify upstream determinants of early-onset risk. If these data are verified and true, the continued rise in cancer incidence will pose an increasing burden on young, reproductive-aged women, reinforcing the need for continued re-evaluation of prevention and screening efforts.

### 4.5. Strengths and Limitations

This study has several strengths. The databases used are large and are representative of the population in the United States. The USCS database provides comprehensive cancer registry data from all 50 states, while NHANES offers continuous standardized data to track population-level lifestyle trends over time. The limitations of this study include its retrospective nature, and inability to control for potential confounding factors such as, stage, histology, and treatment type. As an ecological study, the findings are subject to ecological fallacy, and associations at the group level may not reflect individual-level risk or causation. Because datasets were not linkable at an individual level, we could not evaluate combined effects of multiple lifestyle factors, nor could we adjust for important confounders including diabetes, parity, or exogenous hormone exposure. Obesity was measured using BMI at a single time point, which may not reflect lifetime risk exposure. Several exposures, including dietary intake, physical activity, smoking, and alcohol use, were derived from self-reported survey data and subject to recall and social desirability bias, which may attenuate population-level associations in ecological analyses. The absence of consistent population-level correlations with these variables should therefore not be interpreted as evidence against established individual-level risk relationships reported in cohort studies. Because NHANES data are collected biennially and applied equally across two years, this assumption may introduce bias. The analysis can only determine whether the observed trends in obesity and cancer incidence increase in parallel, without establishing causality. Furthermore, the associations were examined within the same time period, yet cancer may have a substantial latency period. Lastly, the use of multiple unliked datasets precludes the authors from establishing a temporal relationship between obesity and cancer in this population.

## 5. Conclusions

Despite these limitations, this ecological analysis demonstrates that obesity prevalence and the incidence of breast, colorectal, and uterine cancers have increased among young, reproductive-aged women. A graded population-level temporal correlation was seen between obesity class prevalence and cancer incidence trends across all three cancer types.

## Figures and Tables

**Table 1:** Average Annual Percent Change (AAPC) in Cancer Incidence Rates Among Females Aged 20–49, Stratified by Cancer Type, Race/Ethnicity, and Age Group (2001–2018).

Racial Group	Age Group	Breast AAPC for Cancer Incidence (95% CI); *p*-Value	Colorectal AAPC for Cancer Incidence (95% CI); *p*-Value	Uterine AAPC for Cancer Incidence (95% CI); *p*-Value
All	All (20–49)	0.44 (0.3, 0.6); <0.001	1.75 (1.4, 2.1); <0.001	1.64 (1.4, 1.9); <0.001
	20–24	1.69 (1.0, 2.4); <0.001	6.92 (4.3, 9.6); <0.001	2.74 (1.0, 4.6); =0.002
	25–29	1.62 (1.20, 2.1); <0.001	4.15 (3.2, 5.2); <0.001	3.25 (2.4, 4.1); <0.001
	30–34	0.69 (0.30, 1.1); =0.003	2.95 (2.2, 3.7); <0.001	2.92 (2.5, 3.4); <0.001
	35–39	0.20 (0.0, 0.4); 0.03	2.3 (1.9, 2.7); <0.001	2.8 (2.4, 3.2); <0.001
	40–44	0.51 (0.30, 0.7); <0.001	1.67 (1.4, 2.0); <0.001	1.91 (1.4, 2.5); <0.001
	45–49	0.42 (0.3, 0.6); <0.001	0.98 (0.6, 1.3); <0.001	0.78 (0.5, 1.1); <0.001
White	All (20–49)	0.62 (0.5, 0.8); <0.001	2.27 (1.8, 2.8); <0.001	-
	20–24	1.99 (1.0, 2.9); <0.001	7.97 (4.6, 11.4); <0.001	-
	25–29	2.02 (1.3, 2.8); <0.001	4.39 (3.0, 5.8); <0.001	2.49 (1.7, 3.3); <0.001
	30–34	0.83 (0.4, 1.3); <0.001	3.12 (2.4, 3.9); <0.001	1.8 (1.1, 2.5); <0.001
	35–39	0.34 (0.2, 0.5); =0.001	2.86 (2.1, 3.6); <0.001	2.2 (1.7, 2.8); <0.001
	40–44	0.71 (0.5, 0.9); <0.001	2.26 (1.8, 2.7); <0.001	1.42 (0.9, 2.0); <0.001
	45–49	0.6 (0.4, 0.7); <0.001	1.48 (0.9, 2.0); <0.001	0.42 (0.2, 0.6); <0.001
Black	All (20–49)	0.31 (0.1, 0.5); =0.001	-	-
	20–24	1.45 (−0.3, 3.3); 0.11	-	-
	25–29	0.54 (−0.2, 1.3); 0.17	-	2.49 (1.7, 3.3); <0.001
	30–34	0.19 (−0.3, 0.7); 0.49	3.03 (1.9, 4.3); <0.001	1.8 (1.1, 2.5); <0.001
	35–39	0.21 (−0.2, 0.6); 0.28	1.73 (1.0, 2.5); <0.001	2.2 (1.7, 2.8); <0.001
	40–44	0.51 (0.1, 0.9); =0.007	0.32 (−0.4, 1.0); 0.37	1.42 (0.9, 2.0); <0.001
	45–49	0.35 (0.1, 0.6); 0.02	−0.48 (−0.9, −0.0); 0.04	0.42 (0.2, 0.6); <0.001
Hispanic	All (20–49)	-	-	-
	20–24	-	-	-
	25–29	1.17 (0.6, 1.7); <0.001	5.35 (2.7, 8.1); <0.001	4.8 (1.8, 7.8); =0.002
	30–34	0.86 (0.2, 1.6); 0.02	3.23 (0.9, 5.6); 0.01	4.78 (3.9, 5.7); <0.001
	35–39	0.22 (−0.3, 0.7); 0.41	1.68 (−0.1, 3.6); 0.07	3.95 (3.1, 4.8); <0.001
	40–44	0.33 (−0.0, 0.7); 0.07	1.85 (0.8, 2.9); <0.001	2.64 (1.9, 3.4); <0.001
	45–49	0.29 (−0.0, 0.5); 0.08	0.72 (−0.3, 1.8); 0.18	1.66 (0.7, 2.6); <0.001

Values reflect AAPC with 95% confidence intervals and corresponding *p*-values testing whether the AAPC differs from zero within each subgroup (Joinpoint regression), derived from USCS Data. ‘All’ denotes pooled incidence across all racial groups. A dash (-) indicates that data were unavailable or not calculated due to insufficient case numbers.

**Table 2:** Trends in Obesity, Smoking and Alcohol Use Among Women Aged 20–49 years (2001–2018).

Age Group	Obesity (BMI ≥ 30) AAPC (95% CI, *p*-Value)	Smoking AAPC (95% CI, *p*-Value)	Alcohol AAPC (95% CI, *p*-Value)
All (20–49)	1.49 (0.9, 2.1), ***p* < 0.001**	−4.84 (−6.7, −2.9), ***p* < 0.001**	1.45 (0.4, 2.5), ***p* = 0.005**
20–24	1.29 (−0.8, 3.4), *p* = 0.19	−6.02 (−8.7, −3.2), ***p* < 0.001**	−0.18 (−5.4, 5.3), *p* = 0.91
25–29	2.08 (0.3, 4.0), ***p* = 0.03**	−4.32 (−8.7, 0.4), *p* = 0.07	3.78 (−3.0, 11.0), *p* = 0.24
30–34	1.17 (0.0, 2.4), ***p* = 0.04**	−5.25 (−10.6, 0.4), *p* = 0.06	3.67 (−0.9, 8.5), *p* = 0.11
35–39	2.82 (0.6, 5.1), ***p* = 0.01**	−5.42 (−10.3, −0.4), ***p* = 0.04**	1.97 (−1.2, 5.3), *p* = 0.20
40–44	1.69 (0.3, 3.1), ***p* = 0.02**	−4.09 (−8.6, 0.5), *p* = 0.08	0.77 (−5.3, 7.2), *p* = 0.75
45–49	0.51 (−0.6, 1.7), *p* = 0.39	−4.65 (−7.3, −1.8), ***p* < 0.001**	0.09 (−3.0, 3.4), *p* = 0.96

Average Annual Percent Change (AAPC) in obesity, smoking, and alcohol consumption among females aged 20–49 years from 2001 to 2018, stratified by age group. Values reflect AAPC with 95% confidence intervals and corresponding *p*-values testing whether AAPC differs from zero within each subgroup, derived from NHANES data. Significant values bolded for clarity.

## Data Availability

Publicly available datasets were analyzed in this study, including United States Cancer Statistics (USCS): https://www.cdc.gov/cancer/uscs/ (accessed on 1 December 2024), National Health and Nutrition Examination Survey (NHANES) 2001–2018 survey cycles using demographic (DEMO), examination (BMX), questionnaire (ALQ, SMQ), and dietary intake (DR1TOT) files: https://wwwn.cdc.gov/nchs/nhanes/ (accessed on 1 December 2024), and the Behavioral Risk Factor Surveillance System (BRFSS): https://www.cdc.gov/brfss/ (accessed on 1 December 2024). No new data were created or generated in this study.

## Supplementary Materials

The following supporting information can be downloaded at https://www.mdpi.com/article/10.3390/cancers18010167/s1, Figure S1: Trends in obesity prevalence among females aged 20–49 years, 2001–2018; Table S1: Distribution of Breast, Colorectal and Uterine Cancer Cases Among Women Aged 20–49 years (2001–2018); Table S2: Trends in Physical Activity, Kilocalories, Saturated Fat, and Fiber Consumption Among Women Aged 20–49 years (2001–2018); Table S3. Unweighted NHANES sample sizes for female BMI measurements by age group and survey cycle (2001–2018).
