# Supplementary material for: Population-Level Trends in Lifestyle Factors and Early-Onset Breast, Colorectal, and Uterine Cancers
Source: Cancers (Basel). 2026 Jan 3;18(1):167. doi: 10.3390/cancers18010167 (PMC12784836; doi:10.3390/cancers18010167)
Supplement: Supplementary file 1 [file cancers-18-00167-s001.zip › cancers-4055711-supplementary.pdf]

Table S1. *Distribution of Breast, Colorectal and Uterine Cancer Cases Among Women Aged 20-49 years (2001-2018)*

Cancer Type	Age Group (years)	Number of Cases	Percentage of Total (%)
Breast	20-85+	4,686,268	100
	20-49	914,659	19.5
	20-24	3,465	0.07
	25-29	20,635	0.44
	30-34	61,221	1.31
	35-39	134,157	2.86
	40-44	272,179	5.81
	45-49	423,002	9.03
Colorectal	20-85+	1,423,948	100
	20-49	144,130	10.12
	20-24	3,026	0.21
	25-29	5,889	0.41
	30-34	11,325	0.80
	35-39	20,724	1.46
	40-44	37,666	2.64
	45-49	65,500	4.60
Uterine	20-85+	996,362	100

	20-49	124,399	12.48
	20-24	978	0.10
	25-29	4,078	0.41
	30-34	10,456	1.05
	35-39	19,706	1.98
	40-44	33,297	3.34
	45-49	55,884	5.61

This table presents the total number of cases for breast, colorectal and uterine cancers among women aged 20-49 years old from 2001-2018, with a breakdown by 5-year age groups, based on data from the USCS database.

Table S2. Trends in Physical Activity, Kilocalories, Saturated Fat, and Fiber Consumption Among Women Aged 20-49 years (2001-2018)

Age Group	Kilocalories AAPC (95% CI, p-value)	Saturated Fat AAPC (95% CI, p-value)	Fiber AAPC (95% CI, p-value)	Physical Activity AAPC (95% CI, p-value)
**All (20-49)**	-0.09 (-0.4, 0.2), p=0.51	0.24 (-0.3, 0.8), p=0.39	0.97 (0.0, 1.9), p=0.05	0.31 (-0.9, 1.5), p=0.61
20-24	-0.34 (-1.3, 0.6), p=0.41	0.18 (-1.2, 1.6), p=0.77	0.75 (-0.9, 2.4), p=0.31	0.35 (-0.8, 1.5), p=0.54
25-29	-0.07 (-0.7, 0.5), p=0.77	0.64 (-0.5, 1.8), p=0.24	1.24 (0.5, 2.0), **p=0.002**	0.34 (-0.7, 1.4), p=0.52
30-34	0.01 (-0.6, 0.7), p=0.98	0.05 (-1.0, 1.2), p=0.95	1.25 (-0.7, 3.2), p=0.19	0.38 (-0.9, 1.6), p=0.52
35-39	-0.42 (-1.2, 0.4), p=0.25	0.03 (-0.4, 0.4), p=0.89	1.03 (-1.4, 3.5), p=0.33	0.30 (-1.1, 1.7), p=0.65
40-44	0.05 (-1.0, 1.1), p=0.92	0.18 (-1.1, 1.4), p=0.79	0.74 (-1.2, 2.8), p=0.39	0.22 (-1.7, 2.1), p=0.81
45-49	0.17 (-0.2, 0.5), p=0.32	0.42 (-0.8, 1.6), p=0.42	0.65 (-1.0, 2.3), p=0.35	0.20 (-1.2, 1.6), p=0.77

Values reflect AAPC with 95% confidence intervals and corresponding p-values testing whether the AAPC differs from zero within each subgroup. Estimates for kilocalories, saturated fat, and fiber were derived from NHANES data, and estimates for physical activity were derived from BRFSS data. Significant values bolded for clarity.

***Table S3*. Unweighted NHANES sample sizes for female BMI measurements by age group and survey cycle (2001-2018)**

	Year								
Age Group	2001-2002	2003-2004	2005-2006	2007-2008	2009-2010	2011-2012	2013-2014	2015-2016	2017-2018
20-24	297	249	302	234	279	248	245	217	192
25-29	270	214	309	202	270	218	221	278	207
30-34	249	235	241	246	266	233	250	247	222
35-39	242	190	184	257	265	232	250	234	226
40-44	233	202	217	241	297	236	296	264	210
45-49	203	181	195	249	285	223	247	247	200

Values represent unweight samples of female NHANES participants with measured BMI, stratified by 5-year age group and survey cycle (2001-2018). These counts reflect the numbers of individuals contributing to age-specific prevalence estimates; weighted analyses were used for all trend and correlation analyses.

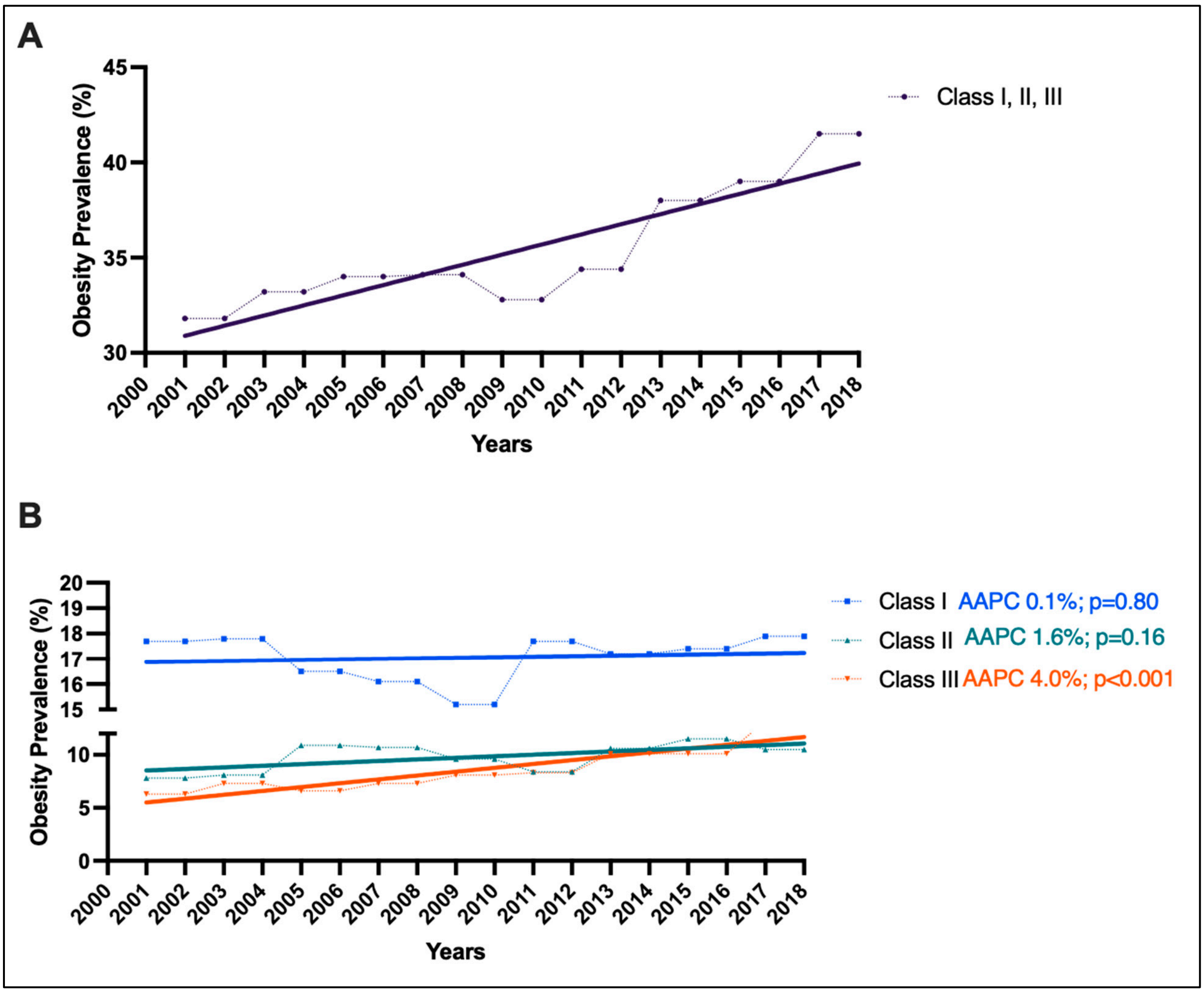

Figure S1A-B. **Trends in obesity prevalence among females aged 20–49 years, 2001–2018.** Trends in obesity prevalence by class among females aged 20-49 years, 2001-2018. Data was obtained from the NHANES database. Figure S1A is a line graph that shows the increasing prevalence of obesity (BMI≥30) over time. Figure S1B demonstrates a similar sentiment however, this graph is stratified by class: Class I (BMI 30-34), Class II (BMI 35-39), Class III (BMI≥40). Class III obesity demonstrated the steepest increase, contributing significantly to the overall rise in obesity prevalence during the study period.
